# A case of complicated parapneumonic pleural effusion caused by paragonimus infection in a child was successfully treated by intrapleural injection of urokinase: a case report and literature review

**DOI:** 10.3389/fped.2025.1557273

**Published:** 2025-06-05

**Authors:** Yangyi Shi, Yan Li, Yu Hu

**Affiliations:** Department of Pediatrics, Mianyang Central Hospital, School of Medicine, University of Electronic Science and Technology of China, Mianyang, China

**Keywords:** child, complicated parapneumonic pleural effusion, paragonimus westermani, urokinase, case report

## Abstract

**Objective:**

The clinical data of a child with complex parapneumonic effusion (PPE) caused by pneumofluke infection were analyzed, and the diagnosis and treatment of the disease were discussed through literature review. The effectiveness and safety of urokinase in the treatment of complex PPE and empyema caused by multiple pathogens were emphasized.

**Methods:**

A 3-year-old male child with pneumofluke infection was admitted to the pediatric department of Mianyang Central Hospital. Chest CT and chest ultrasound showed a right pleural effusion with dense septum formation. The condition was relieved after treatment with praziquantel for anti-infection, thoracic catheter drainage, and urokinase injection into pleural cavity. Through systematic literature search of Pubmed, Embase, CNKI, Wanfang and VIP Chinese databases, no cases of urokinase treatment of pleural effusion caused by pneumofluke infection were found.

**Results:**

A total of 150 ml thick yellow turbidous fluid was drained out of the pleural cavity, and the patient's symptoms and signs were significantly relieved. Reexamination of the chest CT showed that the right pleural effusion was significantly reduced and the right lung was significantly reexpanded. There were no complications such as bronchopleural fistula, pneumothorax, abnormal coagulation function, bleeding and fever during treatment.

**Conclusion:**

Thoracic catheter drainage combined with injection of urokinase is an effective and safe method for the treatment of complex PPE and empyema caused by pneumofluke infection in children. At the same time, the literature review showed that urokinase injection into pleural cavity was effective in the treatment of complex PPE and empyema caused by infection, trauma, tumor and other causes, and no obvious side effects occurred.

## 1 Introduction

Pneumofluke is also known as Paragonimus westermani. Humans were infected mainly by eating raw or semi-raw potamon or crayfish containing cercaria. After infection, adults mainly parasitized in the lung, manifested as cough, sputum, chest pain, etc., which often caused pleural accretion and thickening, and also caused pleural effusion (1). A little pleural effusion may be followed up with anti-infective therapy (2), while a moderate to massive pleural effusion may require concurrent drainage of the pleural cavity. Thoracic catheter drainage alone is not effective for complex PPE and should be treated with fibrinolytic therapy or video-assisted thoracic surgery (VATS) (3). Fibrinolytic therapy is cheaper than VATS, does not require general anesthesia, and is more acceptable to the family of the child. The main drugs used in fibrinolytic therapy include Streptokinase (SK), Urokinase (UK) and Tissue plasminogen activator (t-PA), of which only UK is recommended for children (4). We report a child patient with complex PPE due to pneumofluke infection who was treated with intrapleural injection of UK combined with anti-infective therapy.

## 2 Case presentation

### 2.1 History and physical examination

A male patient, 3 years and 7 months old, developed cough with no apparent cause, accompanied by sputum, occasional nasal obstruction and runny nose on April 5, 2023, and was not treated. On April 6, 2023, he developed a low-to-moderate fever with a maximum body temperature of 38.5° C, wheezing, no shortness of breath, cyanosis, hemoptysis, and no dermatitis, skin nodules or masses. No vomiting, diarrhea, abdominal pain, abdominal distension, headache and other discomfort. The patient's condition did not improve after family members gave the patient oral antipyretic and cough drugs. On April 7, 2023, he was sent to the local hospital for treatment and checked the chest radiograph: increased density in the middle and lower lung field of the right lung, decreased transmittal light, curved dense shadow in the outer band, incomplete display of the right heart margin, and dull right costophrenic Angle. Considering the right lung pneumonia, the right thoracic cavity was likely to be wrapped with fluid. Then he was immediately transferred to our hospital and admitted. About 4 months before admission, the patient had a history of traveling to Chongqing, China, and eating raw pickled river crabs (drunk crabs), and 2 of the other 4 people who ate river crabs together were diagnosed with pneumofluke infection.

Admission for physical examination: Body temperature 38.6℃, pulse 115 times/min, breathing 25 times/min, height 101 cm, weight 16 kg, blood oxygen saturation 96%, clear mind, flushed face, stable breathing, percussion dullness in the middle and lower right lung, thick breathing sound in both lungs, significantly reduced breathing sound in the middle and lower right lung. Moist rales and wheezing sounds were heard in the right upper lung and left lung, and there were no abnormalities in the heart, abdomen and nervous system.

### 2.2 Auxiliary examination upon admission

**Figure 1:**
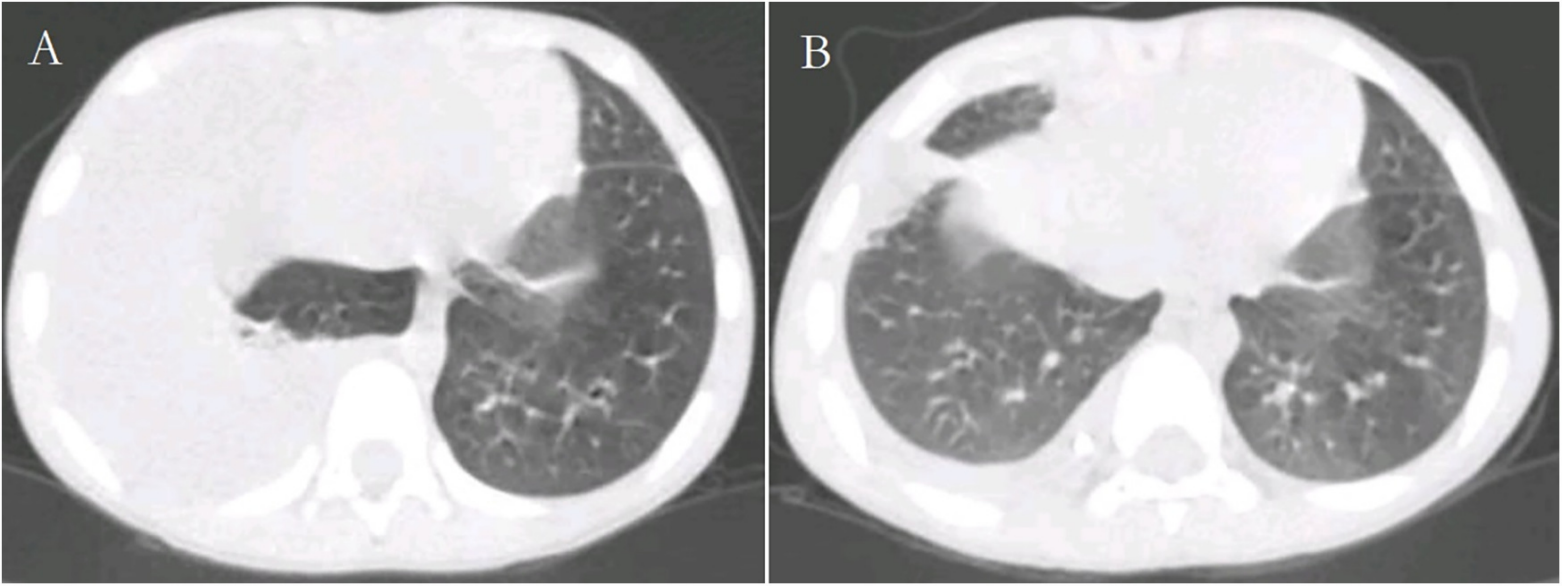
Chest CT (lung windows) of the patient before **(A)** and after **(B)** treatment.

**Figure 2:**
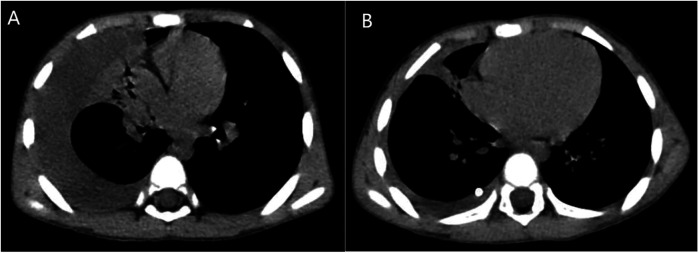
Chest CT (mediastinal windows) of the patient before **(A)** and after **(B)** treatment.

**Figure 3:**
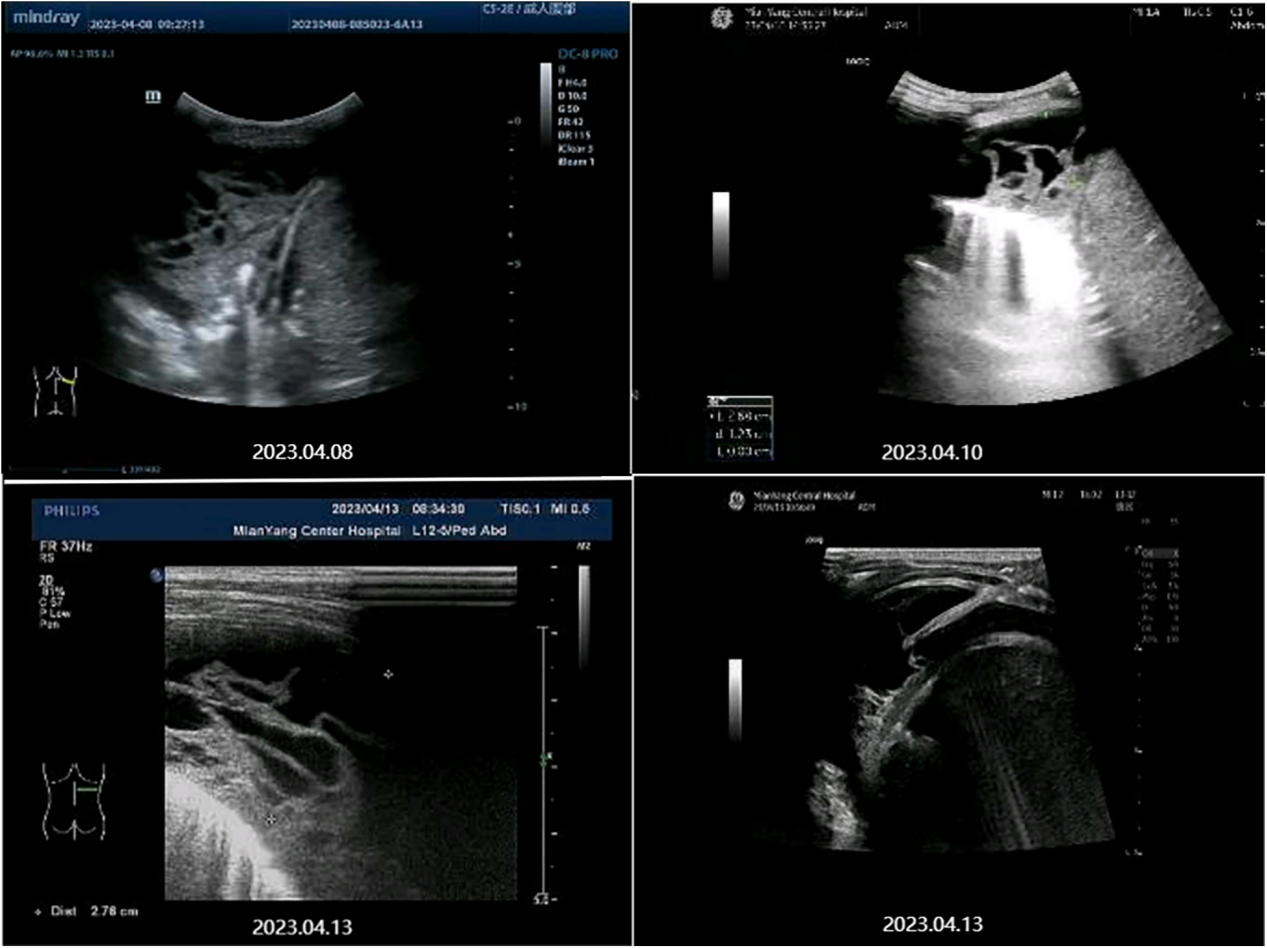
Chest color ultrasound images before treatment.

April 7 Blood analysis and biochemical examination tips: White blood cell (WBC) 15.82 × 10^9^/L↑, Eosinophil (Eos) 7.4 × 10^9^/L↑, Eos% 46.8%↑, C-reactive protein (CRP) 15.33 mg/L↑, Lactate dehydrogenase (LDH) 369 U/L↑, procalcitonin 0.092 μg/L. At the same time, his liver function, kidney function, electrolytes, myocardial markers were normal. Blood immunological examination: IgG 22.6 g/L↑, IgE 4550 IU/ml↑, Mycoplasma pneumoniae IgM 4.52S/CO↑, and Chlamydiae pneumoniae IgM were normal. Influenza A and B virus antigen test, stool and urine routine examination, sputum culture, blood culture and pharyngeal secretion mycoplasma RNA test showed no abnormality. Tuberculin skin test results: 24 h, 48 h, 72 h were negative. Chest CT on April 8 (Figures 1A, 2A): There was moderate fluid accumulation in the right thoracic cavity, partial firmness and atelectasis in the right lung, complete atelectasis in the middle lobe of the right lung, and small nodules in the lower tongue segment of the upper lobe of the left lung. Inflammation was considered. Color ultrasound examination of chest cavity (Figure 3) showed that a dark area of liquid depth of 4.9 cm was visible in the right chest cavity, with dense separation and no safe and effective puncture points, and no obvious dark area of liquid was found in the left chest cavity. Color ultrasound examination of heart, liver, bile, pancreas, spleen and kidney, abdominal cavity and head MRI showed no abnormality.

### 2.3 The patient’s treatment after admission and the changes in his condition

**Figure 4:**
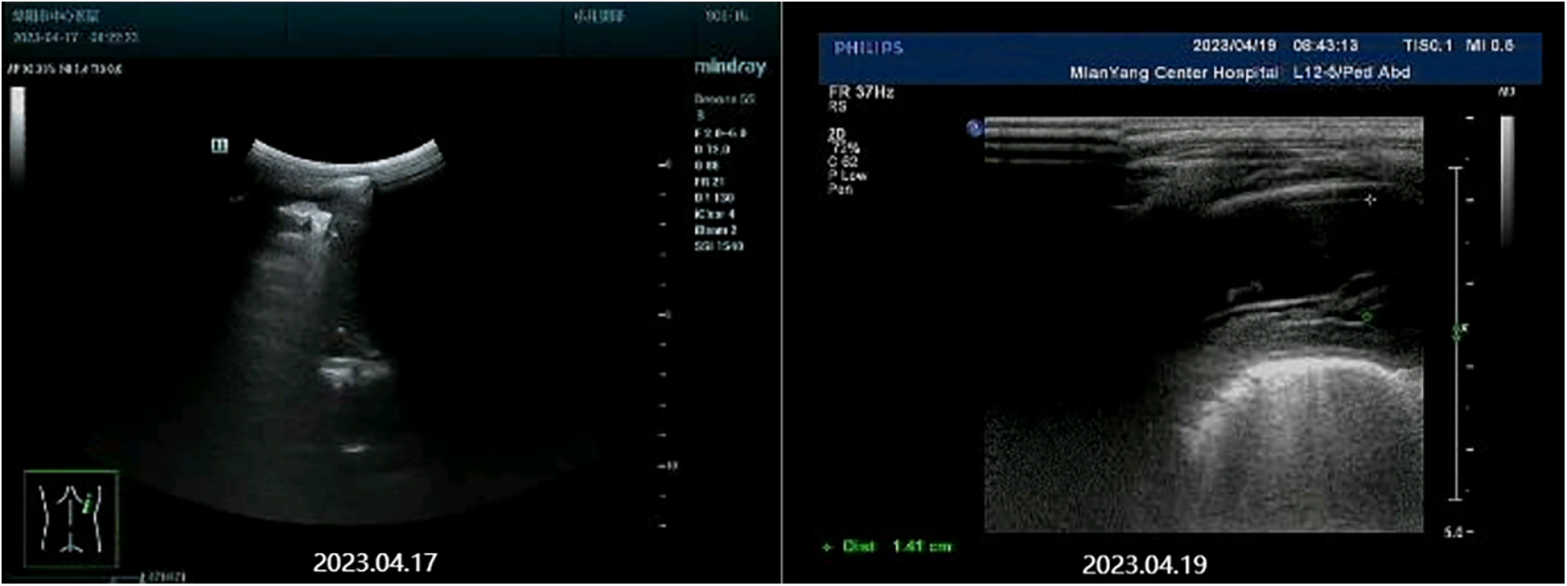
Chest color ultrasound images after treatment.

The patient was diagnosed with asthmatic bronchopneumonia with lung consolidation, atelectasis, and right-sided complex PPE, which could not exclude the possibility of parasitic infection. Treatment began on April 7 with amoxicillin clavulanate potassium and ambroxol hydrochloride intravenous drip, budesonide and salbutamol aerosol. After treatment, the patient's body temperature gradually dropped to normal, there was still cough, wheezing, respiratory sound decreased in the right lung, wet rales and a small amount of wheezing in both lungs. The reexamination of chest ultrasound (Figure 3) on April 10 was slightly better than before: liquid dark area about 2.7 cm deep was visible in the right chest; There was no abnormality in peripheral blood coagulation function (PT, APTT, TT, FIB). Blood analysis:WBC 11.27 × 10^9^/L, Eos 5.4 × 10^9^/L, Eos% 47.9%. On April 12, the patient's lung fluke Antibody IgG test result was positive (blood was taken on admission), and the patient was immediately given praziquantel 400 mg/ time, orally, three times a day, for 3 days (April 12–14). The reexamination of chest ultrasound (Figure 3) on April 13 showed no improvement compared with before: a dark liquid area about 2.8 cm deep was visible in the right chest with dense separation. Therefore, the patient underwent right pleural effusion puncture and catheter drainage under ultrasound guidance. After 80 ml of light yellow fluid is drained, no drainage fluid continues to drain. Results of pleural fluid test: Yellow, slightly cloudy, transparent, no clots, Rivalta test 3+, nucleated cells 2824 × 10^6^/L, red blood cells 3300 × 10^6^/L, mononuclear cells 48%, multiple nuclear cells 52%, LDH 2227U/L, adenosine deaminase 55.1 U/L, total protein 72.18 g/L, glucose 0.06 mmol/L, chloride ion 104.5 mmol/L, no abnormal bacterial culture. The patient was then treated with urokinase, administered at 40,000 U dissolved in 40 ml of normal saline for intrapleural perfusion therapy twice daily (the drainage tube was clamped after each injection, allowing the medicine to remain in the pleural cavity for 4 h before opening the drainage tube), for three consecutive days (from April 13th to 15th). After the first injection of urokinase, 80 ml of thick yellow fluid containing a large amount of flocculent sediment was drained, and the drainage stopped spontaneously. On April 14th, after the injection of urokinase, 110 ml of yellow turbid fluid containing a considerable amount of flocculent material was drained, and the drainage halted naturally. Subsequently, the patient's symptoms and signs significantly improved, with occasional coughing, no wheezing, enhanced respiratory sounds in the right lung compared to before, slightly reduced respiratory sounds, and no dry or wet rales on auscultation of both lungs. On April 15th, 80 ml of yellow turbid fluid was drained after the injection of UK, with a small amount of white flocculent sediment visible inside. A chest CT scan was repeated (Figures 1B, 2B): Compared to the scan on April 8, 2023, the right pleural effusion had significantly reduced, and most of the right lung had re-expanded. Some nodules in the right lung were clearly visible, with a small amount of fluid remaining in the right pleural cavity. There was consolidation and atelectasis in the middle and lower parts of the right lung. Tiny nodules, less than 0.5 cm in diameter, were observed in the inferior lingular segment of the left upper lobe, the posterior segment of the right upper lobe, and the right oblique fissure, which were considered inflammatory or chronic inflammatory. On April 16th, the patient occasionally had mild coughs, with no wheezing or fever. Breath sounds in both lungs were clear and symmetrical, with no wet or dry rales. No further fluid drainage was observed from the drainage tube, and the family members refused further UK treatment, leading to the clamping of the drainage tube. On April 17th, a pleural ultrasound (Figure 4) was repeated, showing a fluid dark area of approximately 0.6 cm deep at the right pleural costophrenic angle. The pleural drainage tube was then removed. On April 19th, a repeat chest ultrasound (Figure 4) was performed, revealing a fluid-filled dark area of approximately 1.4 cm in depth in the right pleural cavity with visible septations. Simultaneously, a blood analysis was conducted which showed WBC 13.59 × 10^9^/L, Eos 6.63 × 10^9^/L, Eos% 48.8%, while CRP and coagulation function were normal. Liver function tests showed albumin 37.15 g/L↓, LDH 324 U/L↑. IgE level was 5,560 IU/ml. The patient had occasional mild cough, no fever, no wheezing, stable breathing, and clear and symmetrical breathing sounds in both lungs, with no wet or dry rales present. As the family members did not consent to continued thoracic drainage, UK therapy, bronchoscopy, or bronchoalveolar lavage, the patient was discharged on April 20th. The discharge diagnosis included Paragonimiasis, Complex PPE, and Asthmatic bronchopneumonia with consolidation and atelectasis.

## 3 Discussion

Pulmonary paragonimiasis is the most common type of paragonimiasis, with 90% of reported cases occurring in Asia, most frequently in Southeast Asia (5), particularly in regions such as China, Korea, and Japan (6). The majority of symptomatic infections manifest as pulmonary diseases, while extrapulmonary manifestations are rare. In early-stage infections, larvae hatch in the small intestine and migrate within the human body through the gastrointestinal wall. During this phase, patients may experience mild gastrointestinal discomfort or skin rashes, or they may remain asymptomatic. Subsequently, the larvae penetrate the diaphragm and move within the pleural cavity, potentially leading to pleural effusion, pneumothorax, and pleural thickening. In late-stage infections, adult worms reside and lay eggs in the lungs or parasitize in ectopic locations, which can give rise to various symptoms and signs of respiratory infections or manifestations of ectopic parasitism. This stage occurs no less than one month or even longer after infection with Paragonimus westermani (6).

Referring to the “Diagnosis of Paragonimiasis: WS 380-2012” (1), the patient meets the following criteria: ① History of eating raw freshwater crabs, and co-diners have been diagnosed with paragonimiasis; ② Clinical manifestations of lung infection including cough, fever, wheezing, lung wheeze, and wet rales; ③ Significant increase in the count and proportion of eosinophils in peripheral blood; ④ Positive serum immunology test; ⑤ Abnormal findings on imaging examinations. Based on these, a clinical diagnosis of paragonimiasis can be made. The confirmation of paragonimiasis relies on positive etiological examination, which involves the detection of Paragonimus eggs or worms in sputum, feces, or other living tissues or body fluids (1, 5). Collecting sputum for 24 h can improve the sensitivity of egg detection. However, due to poor cooperation from the patient's family members and difficulties in collecting sputum, this examination could not be arranged.

The patient's chest CT scan indicated a moderate amount of fluid in the right pleural cavity. According to statistics, over 50% of patients with paragonimiasis present with pleural lesions and pleural effusion (5, 7). The pleural effusion caused by paragonimiasis infection is typically eosinophilic, characterized by a low pH, low glucose level, high LDH, and high protein content (6, 8). Additionally, there is a significant increase in eosinophils in the pleural fluid (8). The patient's pleural fluid analysis revealed the following results: glucose 0.06 mmol/L, LDH 2,227 U/L, Rivalta test 3+, and total protein 72.18 g/L, which are consistent with the characteristics of pleural effusion caused by paragonimiasis infection. Furthermore, the glucose level was <2.2 mmol/L and the LDH was >1,000 U/L, with dense septations visible on ultrasound, providing evidence of complex PPE (9).

Upon literature review, there is a lack of research data on treatment methods and efficacy for complex PPE caused by paragonimiasis infection. In reviewing PPE studies, it is found that most pleural effusions can be alleviated through antibiotic treatment, while approximately 30% of pneumonia cases complicated by pleural effusion require thoracic drainage therapy. The treatment options for complex PPE and pyothorax include antibiotics, chest tube drainage, intrapleural fibrinolytic therapy, VATS, or thoracotomy with pleural fiberboard stripping (10–12). In this patient's case, classified as complex PPE, the administration of amoxicillin and clavulanate potassium was based on the possibility of concomitant bacterial infection. After treatment, the patient's body temperature gradually returned to normal, and the pleural effusion decreased, indicating the effectiveness of the treatment. However, the relief of cough, wheezing, and lung signs was not significant. Upon obtaining a positive IgG antibody test for paragonimiasis, praziquantel was immediately added to the treatment regimen.

In clinical practice, there are significant variations in the treatment approaches for PPE and pyothorax in children. Some scholars advocate for early use of VATS (12) or early surgical debridement (13). According to research statistics from 2018, 50% of patients with pyothorax or PPE underwent thoracotomy (13). However, other scholars prefer initial treatment with thoracic catheter drainage combined with intrapleural fibrinolytic therapy (12). Studies have shown that there are no significant differences in postoperative fever duration, total hospital stay, and months until radiological normalization between thoracoscopic surgery and intrapleural injection of UK for the treatment of complex PPE and pyothorax (12, 14). The overall mortality rate of pleural fibroblastic decortication is 3%, and the incidence of postoperative complications is 39% (15). On the other hand, intrapleural fibrinolytic therapy can reduce the need for surgical intervention (10, 13, 16, 17), avoiding thoracotomy in 77% of patients with complex PPE (18). Considering that there is no significant difference in efficacy between UK treatment and surgery, surgery requires general anesthesia and carries risks of side effects and complications, and fibrinolytic therapy is more economical than surgical treatment (12), we chose thoracic drainage combined with intrapleural fibrinolytic therapy as the initial treatment for this patient's complex PPE.

So far, intrapleural injection of fibrinolytic drugs has been widely used to treat complex PPE and empyema. The use of fibrinolytic drugs can promote fibrin degradation, decompose fibrin clots, reduce the viscosity of pleural effusion, and dissolve fibrous septa, thereby facilitating pleural cavity drainage (19). At the same time, it can clear lymphatic pores, establish effective filtration and reabsorption of pleural fluid function, and restore normal pleural fluid circulation dynamics (4), thereby reducing the need for surgical intervention (19, 20). The British Thoracic Society (BTS) guidelines recommend intrapleural fibrinolysis therapy to shorten hospital stays and recommend it for any complex PPE or empyema (4).

The primary drugs used in fibrinolytic therapy include SK, UK, and t-PA (such as tenecteplase and alteplase). SK, a non-enzymatic streptococcal exotoxin, possesses antigenicity, while UK is a thrombolytic agent extracted from cultured human neonatal kidney cells and lacks antigenicity (21). Studies have indicated no significant differences in the efficacy of SK, UK, and alteplase (16, 22–24). However, UK demonstrates higher safety compared to SK, as SK treatment is associated with a higher incidence of allergic reactions such as fever (22–24). Alteplase shows a higher rate of adverse reactions (16). Early studies indicated that UK therapy had a significant positive effect on reducing surgical intervention, whereas SK and t-PA did not exhibit such an effect (17). However, subsequent research revealed no significant difference in surgical needs between treatment with SK and UK, as well as alteplase and UK (16). Additionally, there was no significant difference in efficacy between UK treatment and VATS (12, 14), but VATS offers a significantly higher success rate in first-time treatment and shorter hospital stays compared to SK (25). Therefore, UK is currently the most commonly used fibrinolytic drug (20). Studies have also suggested that the combination of fibrinolytic agents with DNase can achieve better treatment outcomes compared to the use of UK, SK, or t-PA alone. However, these findings are limited to animal experiments and adult studies, and there is a lack of research on the use of DNase in the pleural cavity of children (26, 27). Meanwhile, we have found a case report describing a severe complication of hemothorax with White-Out Lung during the treatment of complex PPE using a combination of t-PA and DNase in the pleural cavity (28). The BTS guidelines recommend UK only for the treatment of complex PPE and empyema in children (4). Therefore, we chose to use UK for this patient.

UK has been proven to be safe and effective in the treatment of complex PPE for adults (10, 16, 19, 22, 23, 29–31), without a significant increase in severe side effects (17) or significant differences in the risk of death. Additionally, no other complications caused by the treatment have been reported (16). Similarly, UK has been found to be safe and effective in the treatment of complex PPE and empyema for children (4, 20, 32–45), with no reported complications such as pain, fever, intrapleural bleeding, pneumothorax, or systemic bleeding (30, 46). UK therapy can shorten the duration of hospital stay and reduce the need for pleural debridement (46). Over 90% of children with complex PPE can be treated with chest tube drainage combined with UK injection, obviating the need for surgical intervention (36, 38). Following the BTS guidelines, the patient in this case was treated with UK 40,000 U diluted in 40 ml of normal saline, administered via intrapleural infusion twice daily for three consecutive days. Before the intrapleural infusion of UK, only 80 ml of pale yellow fluid was drained, and no further fluid was drained subsequently. However, after the infusion, a total of 150 ml (excluding the infused drug volume) of thick yellow turbid fluid containing a large amount of flocculent material was drained, indicating empyema based on the fluid's characteristics. Three days after the intrapleural infusion of UK, the patient's symptoms and signs were significantly relieved. A repeat chest CT scan showed a significant reduction in right pleural effusion and substantial re-expansion of the right lung (as shown in Figure 1B), demonstrating a remarkable therapeutic effect. No adverse reactions such as chest pain, pneumothorax, fever, rash, or bleeding occurred during the treatment process.

According to existing reports, complex PPE and pyothorax caused by various etiologies can be effectively treated through intrapleural injection of fibrinolytic agents. A total of 40 relevant studies were retrieved, including 22 studies on adults and 18 studies on children. The most studied condition was encapsulated pleural effusion or pyothorax caused by Mycobacterium tuberculosis infection (19 studies). Among other studies on the treatment of complex PPE and pyothorax with UK, the most commonly detected pathogenic bacterium was Staphylococcus aureus (16 studies), followed by Streptococcus pneumoniae (13 studies), Haemophilus influenzae, Escherichia coli, and Group A Streptococcus (7 studies each), Pseudomonas aeruginosa (5 studies), Peptostreptococcus (4 studies), Mycoplasma pneumoniae and Enterobacter (3 studies each), Klebsiella pneumoniae, Streptococcus pyogenes, Bacteroides ureolyticus, and Fusobacterium (2 studies each), as well as Group B Streptococcus, Acinetobacter, Bacteroides fragilis, Haemophilus parainfluenzae, Pseudomonas, Raoultella ornithinolytica, Klebsiella, Streptococcus, Morganella, Streptococcus milleri, Bacteroides, Staphylococcus, Proteus mirabilis, Staphylococcus hominis, and Candida albicans (1 study each). Other etiologies included malignant pleural effusion caused by tumors (4 studies) and encapsulated pleural effusion after thoracic surgery (1 study) (20, 22, 29–39, 41, 43–68). No relevant treatment literature was found for complex PPE caused by parasitic infections. This article reports a case of complex PPE in a child caused by Paragonimus infection. Thoracic ultrasound showed the formation of fibrous septations with no effective puncture site. After ultrasound-guided thoracic catheter placement and drainage of 80 ml of pale yellow fluid, further smooth drainage could not be achieved. The patient's condition improved after intrapleural infusion therapy with UK, and no adverse reactions occurred during the treatment process. This suggests that this therapy may be equally safe and effective for complex PPE caused by parasitic infections, expanding the range of indications for intrapleural infusion therapy with UK.

## 4 Conclusion

In summary, paragonimiasis can lead to the formation of complex PPE and pyothorax. The confirmation of paragonimiasis relies on positive etiological examination results. The limitation of the case reported in this article is that it was only clinically diagnosed as paragonimiasis without etiological examination. Clinicians should promptly collect 24-hour sputum, pleural fluid, or other bodily fluids or biopsy samples from suspected paragonimiasis cases to detect Paragonimus eggs or worms for confirmation. The efficacy of thoracic drainage alone is poor for complex PPE. For complex PPE caused by paragonimiasis, intrapleural injection of UK combined with chest tube drainage may be an effective and safe treatment option. Literature review indicates that intrapleural injection of UK combined with thoracic drainage is effective for the treatment of complex PPE and pyothorax caused by various etiologies, with no significant adverse reactions such as fever, pneumothorax, or bleeding. It is possible that UK therapy could be safely and effectively utilized for pediatric patients with complex PPE and empyema, regardless of the underlying cause.

## Data Availability

The original contributions presented in the study are included in the article/Supplementary Material, further inquiries can be directed to the corresponding author.
